# Dr. William Merwin Smith

**Published:** 1902-02

**Authors:** 


### OBITUARY.

Dr. William Merwin Smith, former health officer of the port of New York, died in Redlands, Cal., January 10, 1902, in his 76th year. He was born in Paterson, N.J., his English and Dutch ancestors having come to this country in the early part of the sixteenth century. His father and grandfather were surgeons, and besides being distinguished in medicine he had an active political career. He was twice elected to the legislature, was several times a delegate to National conventions, notably when Lincoln was nominated. He was surgeon-general on Governor Dix's staff, and served during the war as surgeon of the Third Brigade, Casey's division in the Army of the Potomac.

Dr. Smith was appointed health officer of the port of New York by Governor Cornell in 1880, he then being a resident of Allegany County, and held it longer than any other incumbent.

He was an able and efficient health officer for twelve years, and in his administration many reforms were carried out. During his term it was made a salaried office.

Dr. Smith lived in Brooklyn after his retirement from office and became an active student of bacteriology and natural history, having been a collector in the latter branch, as well as a student. In the last few years he had lived in Redlands, Cal. He leaves a wife and two sons--Frank Sullivan Smith, a lawyer of New York, and Clarence Melbury Smith, also a lawyer, living in Redlands.

